# Effects of exposure to *Streptococcus iniae* on microRNA expression in the head kidney of genetically improved farmed tilapia (*Oreochromis niloticus*)

**DOI:** 10.1186/s12864-017-3591-z

**Published:** 2017-02-20

**Authors:** Jun Qiang, Fanyi Tao, Jie He, Lanyi Sun, Pao Xu, Wenjin Bao

**Affiliations:** Key Laboratory of Freshwater Fisheries and Germplasm Resources Utilization, Ministry of Agriculture, Freshwater Fisheries Research Center, Chinese Academy of Fishery Sciences, 9 Shanshui East Road, Wuxi, Jiangsu 214081 China

**Keywords:** GIFT, microRNA, Deep sequencing, Target prediction, *Streptococcus iniae*

## Abstract

**Background:**

Genetically improved farmed tilapia (GIFT, *Oreochromis niloticus*) are susceptible to infection by *Streptococcus iniae* when maintained in modern intensive culture systems. GIFT are commercially important fishes that are cultured widely in southern China. The role of microRNAs (miRNAs) in the regulatory response of GIFT to *S. iniae* infection has been underestimated and has not yet been well studied. Head kidney has an important immune function in teleost fishes. The main aim of this study was to determine the possible function of miRNAs in head kidney of *S. iniae*-infected GIFT. MiRNAs are small, non-coding RNAs that regulate gene expression by binding to the 3’-untranslated regions of their target mRNAs. MiRNAs are known to regulate immune-regulated signaling and inflammatory response pathways.

**Results:**

High-throughput deep sequencing of two libraries (control group [CO] and infected group [IN]) of RNA extracted from GIFT head kidney tissues generated 12,089,630 (CO) and 12,624,975 (IN) clean reads. Bioinformatics analysis identified 1736 and 1729 conserved miRNAs and 164 and 165 novel miRNAs in the CO and IN libraries, respectively. Three miRNAs (miR-310-3p, miR-92, and miR-127) were found to be up-regulated and four miRNAs (miR-92d-3p, miR-375-5p, miR-146-3p, and miR-694) were found to be down-regulated in the *S. iniae*-infected GIFT. The expressions of these miRNAs were verified by quantitative real-time PCR. RNAhybrid and TargetScan were used to identify complementary miRNA and mRNA target sites, and the Gene Ontology and Kyoto Encyclopedia of Genes and Genomes databases were used to annotate and predict potential downstream regulation of biological pathways. Seven target genes, which encode immune-related proteins (complement C3, cytidine deaminase, regulator of G-protein Rgs22, mitogen-activated protein kinase Mapk1, metabotropic glutamate receptorm GluR8, calcium-sensing receptor CaSR, and microtubule-associated protein Map1S) were predicted to play crucial roles in the GIFT response to *S. iniae* infection.

**Conclusions:**

*S. iniae* outbreaks have hindered the development of the tilapia industry in China. Understanding the miRNA transcriptome of *S. iniae*-infected GIFT is important for exploring the immune responses regulated by miRNAs as well as for studying novel regulated networks to prevent and treat *S. iniae* infections in the future.

**Electronic supplementary material:**

The online version of this article (doi:10.1186/s12864-017-3591-z) contains supplementary material, which is available to authorized users.

## Background

Genetically improved farmed tilapia (GIFT, *Oreochromis niloticus*) is a commercially important farmed fish in southern China [1]. Data from the Food and Agriculture Organization show that tilapia output reached 5,446,800 tons in about 100 farming countries during 2014. In China, the tilapia output was estimated to be about 145 million tons in 2014. Because of its intraspecific cross and relatively rapid growth rate, GIFT is highly susceptible to bacterial diseases triggered by *Streptococcus iniae* and *Streptococcus agalactiae* when reared in intensive culture systems [2]. Thus, a better understanding of the regulatory response of GIFT to infection will provide crucial information for both immunomodulation and GIFT breeding.

The non-specific immune system in fish plays a major role at all stages of infection [3]. Thus, understanding the immune regulation mechanism may contribute to better disease prevention. MicroRNAs (miRNA) are small non-coding single-stranded RNAs (18–25 nucleotides) that are found in plants, animals and some viruses. MiRNAs function in RNA silencing and post-transcriptional regulation of gene expression by binding to the 3’-untranslated regions of their target mRNAs [4]. MiRNA-mediated regulation has been reported in the innate immune response, inflammatory diseases, cell signal transduction, and targeted therapy [5]; however, the functions of miRNAs have been studied mainly in mammalian and plants [6, 7]. The miRBase database (Release 21.0, June 2014) (http://www.mirbase.org/) contains 28,645 miRNAs in 223 species expressing 35,828 mature miRNAs. Many mature miRNAs are highly conserved across vertebrate taxa [8], but their role in the fish host immune response is known in only a few species, mainly zebrafish (*Danio rerio*) [9], large yellow croaker (*Pseudosciaena crocea*) [10], and grass carp (*Ctenopharyngodon idella*) [11]. Several immune-specific miRNAs that regulate innate and adaptive immune responses in fish have been identified and classified, including miR-122, miR-192, miR-194a, miR-146a/b, and miR-155. For example, miR-122 and miR-194a were markedly decreased in zebrafish infected by *Vibrio harveyi*, and miR-146a/b and miR-155 down-regulated Toll-like receptor 4 (TLR4) and downstream nuclear factor kB activity in zebrafish with lipopolysaccharide infection [9]. Up-regulated expression of miR-122-3p, miR-214-3p, and miR-181c was detected in infected large yellow croaker treated with polyriboinosinic: polyribocytidylic acid (poly[I:C]) [10]. Further, let-7i and miR-118 were reported to down-regulate TLR4 and nuclear factor interleukin 3-6 (NFIL3-6) in response to an excessive inflammatory response in grass carp infected with *Aeromonas hydrophila* [11]. Aberrant regulation of some of these miRNAs can disrupt intracellular signaling networks and inflammatory responses, which can result in pathological conditions [12].

In recent years, a growing number of genes related to immunology have been detected and identified in tilapia using various molecular biological techniques [3]. Following the successful completion of the *O.niloticus* genome project (http://www.ncbi.nlm.nih.gov/genome/?term=Oreochromis%20niloticus), studies on the expression and regulation mechanisms of miRNA-mediated genes in the immune response will grow in importance. However, *O. niloticus* miRNA transcriptome data have not yet been reported, and changes in miRNA expression profiles in the GIFT immune response to a foreign challenge have not been studied until now. The aim of this study is to identify and analyze the miRNAs and their target genes involved in the immune response of GIFT to *S. iniae* infection. Two small RNA libraries from GIFT infected with *S. iniae* for 24 h and uninfected GIFT were sequenced by high-throughput sequencing. MiRNAs were identified and RNAhybrid and TargetScan were used to predict miRNA target genes, and the Gene Ontology (GO) and Kyoto Encyclopedia of Genes and Genomes (KEGG) databases were used to explore potential biological processing of downstream targets genes. We identified miRNAs and their target genes involved in the immune response and verified the expression profiles of miRNAs by quantitative real-time PCR (qRT-PCR). Identification of miRNAs involved in immune regulation is an essential prerequisite towards better understanding of their function in intracellular signaling networks and inflammatory responses. The new insights from this study will pave the way for the development of disease control measures and for genetic improvement in GIFT.

## Results and discussion

Streptococcal disease caused by *S. iniae* is unquestionably one of the main bacterial diseases in tilapia. It has been reported to cause huge mortality and economic loss in tilapia culture zones in more than four provinces in China, including Guangdong, Guangxi, Hainan, and Fujian. However, very few studies have described the molecular mechanisms underlying the regulation of the miRNA-mediated immune response in tilapia. The pre-experiment determined that the 96 h semi-lethal *S. iniae* dose (96 h LD_50_) in GIFT was 10^7^CFUmL^-1^ (Additional file 1: Table S1). GIFT infected with a concentration of 10^7^CFUmL^-1^ could stimulate their own immune systems to deal with bacterial invasion 24 h post- *S. iniae* infection [2, 13], the survival rate was 90%. In a previous study, we found that C-type lysozyme, heat-shock protein 70 (Hsp70), interleukin-1β (IL-1β), interferon-γ (IFN-γ), and tumor necrosis factor-α (TNF-α) expression levels in the head kidney tissue of *S. iniae*-infected GIFT for 96 h reached peak values 24 h post-infection [2]. However, prolonging the infection time weakened the fish’s own immune response, survival decreased rapidly at 36 h and 48 h post-infection in the pre-experimental group injected with 10^7^ CFUmL^-1^. The following symptoms occurred after 24 h in GIFT infected with the 96 h LD_50_, diseased fish began to rotate intermittently in the water, unbalanced swimming motions, corneal opacity in the white of the eye, enlarged and reddened spleen, liver congestion, hemorrhaging kidneys, enlarged gallbladder, and dark green bile. Hemorrhaging kidneys may have resulted from lymphatic cell reduction and replacement by a large number of red blood cells, and a high rate of macrophage cell infiltration [14]. Macrophages can reduce bacterial invasion by phagocytosis. Head kidney was found to be an important immune-regulated tissue in adult fish during the early stage of bacterial infections [15], and is considered to play a similar role as bone marrow in mammals [16]. Head kidney is composed mainly of granulocytes, macrophages, and T and B lymphocytes in the presence of any acquired specific or non-specific immunoregulation [17]. Therefore, in this study, we aimed to identify miRNAs involved in resistance to pathogens infection and miRNA-mediated regulatory pathways, to better understand the immune-regulated response in tilapia head kidney triggered by a *S. iniae* challenge.

Two small RNA libraries from the head kidney of GIFT infected with *S. iniae* for 24 h (IN) and from uninfected GIFT (CO) were built and sequenced. A total of 12,423,338 and 12,845,540 raw reads were obtained from the CO and IN libraries, respectively (Table 1). After removing reads that contained adaptor sequences and low-quality reads, 12,089,630 and 12,624,975 clean reads remained in the CO and IN libraries, respectively. We found that 38.63% (CO) and 39.16% (IN) of the reads had no matches in the searched databases; these unannotated sequences were considered to be unique reads. The total clean reads contained 72.08% (CO) and 64.36% (IN) of the conserved miRNAs, and the unique reads contained 13.94% (CO) and 11.96% (IN) of the conserved miRNAs (Additional files 2 and 3: Tables S2 and S3).

**Table 1:** Preliminary analysis of solexa high-throughput sequencing data

	CO library	IN library
Raw reads	12,423,338	12,845,540
Clean reads	12,089,630	12,624,975
Total small RNA(18–30 nt)	541,895	640,740
Mapping to genome	264,661	312,600
Known miRNA	1736	1729
Novel miRNA	164	165

**Fig. 1:**
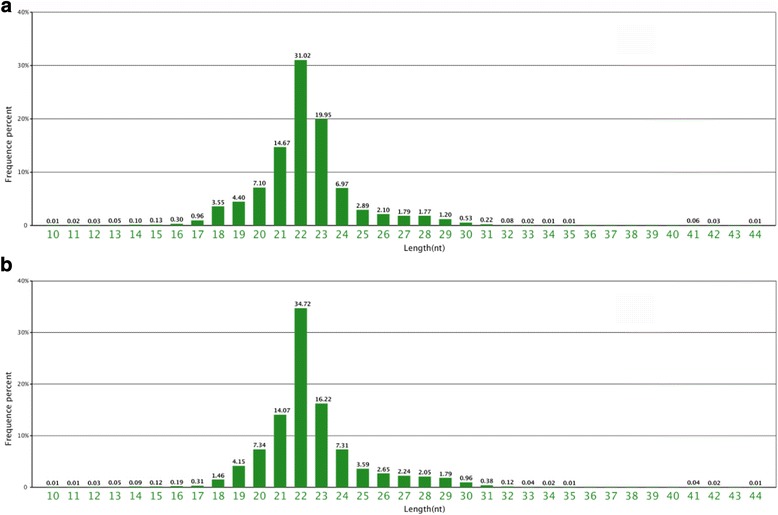
Length size distribution small RNA sequences both the control group (**a**) and infected group(**b**)

We found that 264,661 (CO) and 312,600 (IN) of the unique reads and 164 and 165 of the candidate novel miRNAs could be mapped to the *O. niloticus* (Nile tilapia) genome sequence. The lengths of the read sequences in the two libraries ranged from 10 nt to 44 nt, with the 22-nt, 21-nt, and 23-nt long reads being the most abundant (Fig. 1). Li et al. [18] and Hong et al. [19] also reported that 22-nt long miRNAs were enriched in miRNA transcriptome libraries of both *Apostichopus japonicus* and *Gobiocypris rarus*.

**Fig. 2:**
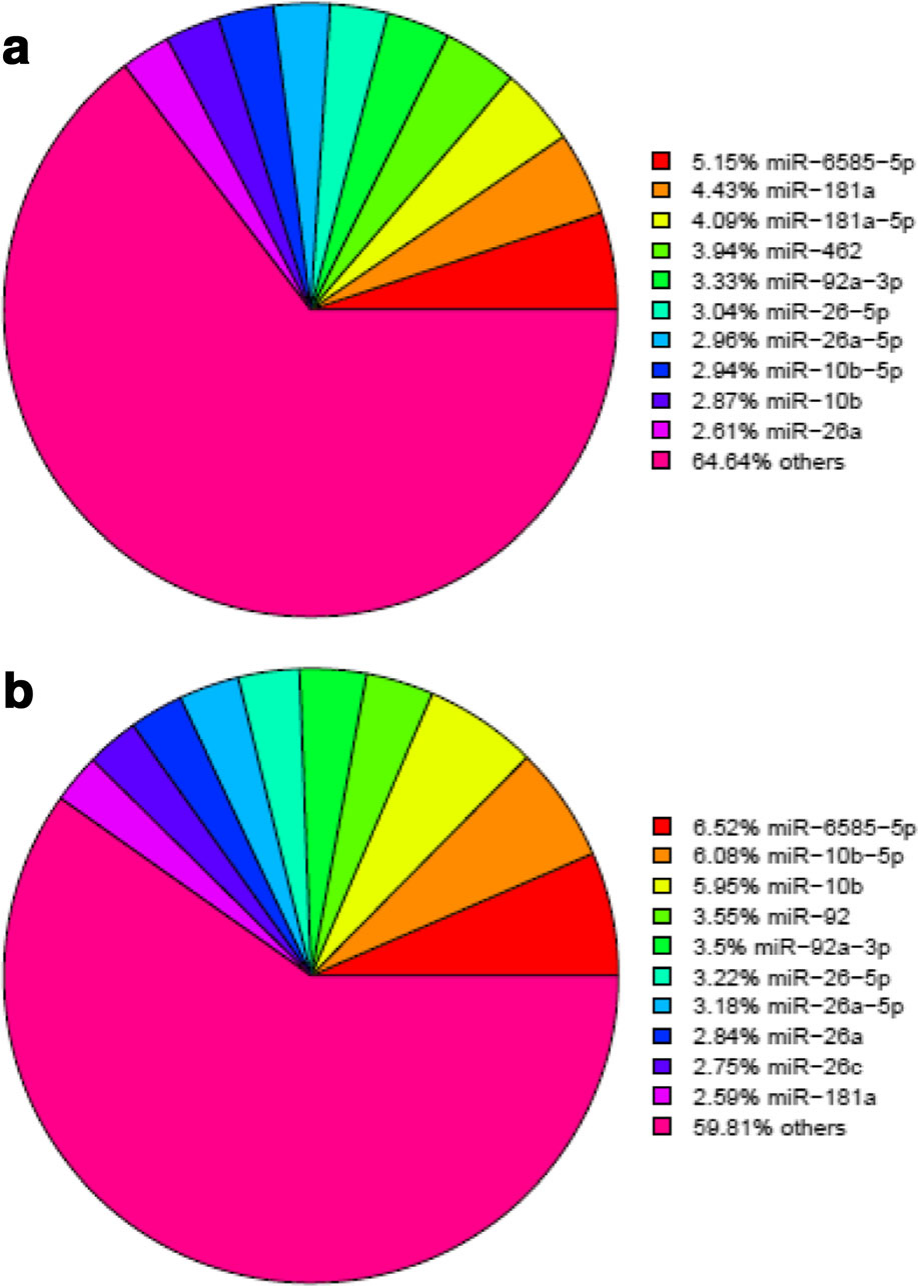
Top 10 lists of most abundant miRNAs both the control group (**a**) and infected group (**b**)

The numbers of reads in a library have been employed to estimate the abundance of the reads in a sequencing sample. The ten most abundant miRNAs in the CO library were miR-6585-5p, miR-181a, miR-181a-5p, miR-462, miR-92a-3p, miR-26-5p, miR-26a-5p, miR-10b-5p, miR-10b, and miR-26a (Fig. 2a), and they accounted for 35.4% of the reads that mapped to sequences in miRBase. Eight of these miRNAs were among the ten most abundant miRNAs in the IN library (miR-6585-5p, miR-181a, miR-92a-3p, miR-26-5p, miR-26a-5p, miR-10b-5p, miR-10b, and miR-26a) (Fig. 2b), which accounted for 40.2% of the reads that were mapped to sequences in miRBase. Among these miRNAs, miR-181a, miR-92a-3p, miR-26a-5p, miR-10b, and miR-26a have been reported to play fundamental roles in immune-regulated signaling and inflammatory responses in human [20–24].

**Fig. 3:**
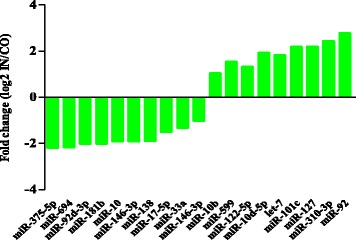
Fold changes of differentially expressed miRNAs between the control group (CO) and infected group (IN). Fold changes were calculated as log2 (IN/CO)

**Fig. 4:**
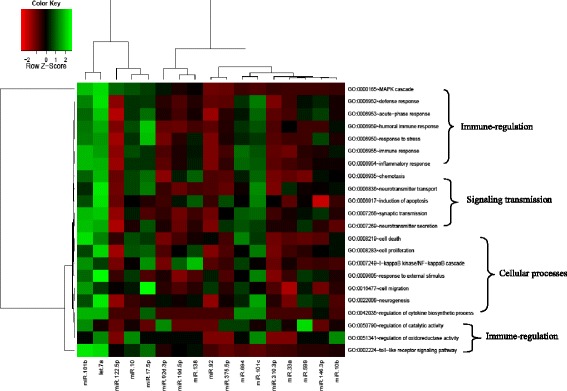
Cluster analysis of differentially expressed miRNAs. The *color* indicates the log2-fold change from high (*red*) to low (*green*), as indicated by the color scale. The names of the miRNAs and the clusters to which they belong are shown in the bottom of the panel. A heat map was constructed based on the 22 biological processes of the miRNA target genes indentified by a gene ontology analysis

We selected and analyzed 19 differentially expressed miRNAs with more than 10 reads between the CO and IN libraries (Additional file 4: Table S4). Among the 19 miRNAs, eight (miR-92, miR-101c, miR-310-3p, miR-127, let-7, miR-599, miR-122-5p, miR-10d-5p) were significantly up-regulated and 11 (miR-92d-3p, miR-146-3p, miR-17-5p, miR-10, miR-694, miR-181b, miR-138, miR-10-5p, miR-33a, miR-375-5p, miR-10b) were significantly down-regulated in the IN library compared with the CO library (Fig. 3). We screened for putative miRNA target genes using RNAhybrid and TargetScan with the *O. niloticus* genome sequence. The candidate target genes were annotated with GO terms, and 22 biological processes were assigned to the predicted targets (Fig. 4). The heat map indicates that these differentially expressed miRNAs may play important roles in immune-regulation, signaling transmission, and cellular processes (Fig. 4).

**Fig. 5:**
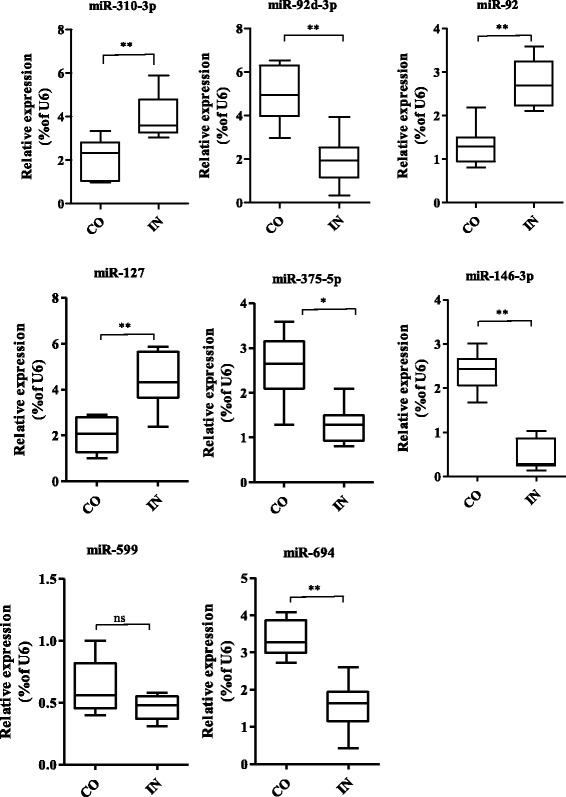
Quantitative real-time PCR validation of eight miRNA expression levels both the control group (CO) and infected group (IN). The levels of the indicated miRNAs expression were showed by Box and whisker plots (*n* = 10 replicates per group). The values are expressed as the relative ratio with U6 as an internal control. *P < 0.05 and **P < 0.01 by unpaired Student’s T-tests. NS, no significant difference

To verify the expression levels estimated from the transcriptome sequencing data, we measured the expression levels of eight miRNAs (miR-92d-3p, miR-127, miR-310-3p, miR-146-3p, miR-599, miR-375-5p, miR-92, and miR-694) by qRT-PCR. The eight miRNAs were selected because they had relatively high fold-changes post-infection. The PCR results showed significant up-regulation of miR-310-3p, miR-92, and miR-127 and significant down-regulation of miR-92d-3p, miR-375-5p, miR-146-3p, and miR-694 in the IN group compared with the CO group (Fig. 5), in agreement with the results obtained from the transcriptome data. However, the PCR results did not show up-regulation of miR-599 expression in the IN group (Fig. 5), which contradicts the result obtained from the transcriptome data. In our study, we considered transcripts with more than 10 reads; however, Cristino et al. [25] considered that transcripts with less than 100 reads may be unreliable and only roughly measure relative miRNA abundance. The miR-599 transcript in the CO library was represented by 41 reads, which was the lowest number of reads among the eight selected miRNAs (Additional files 4: Table S4). This might explain the difference between the miR-599 expression levels estimated from the transcriptome sequencing data and by qRT-PCR.

Previous observations have suggested that miR-146, miR-127, miR-92a, miR-310, miR-375, and miR-694 play vital immune-regulated functions in fish and mammals. For example, a recently study by Zhang et al. [26] found that miR-92a was up-regulated in sea cucumber exposed to *Vibrio splendidus* and lipopolysaccharides and its target gene was predicted as E3 ubiquitin-protein ligase (SMURF), which might act as a tumor suppressor by modulating the signaling pathway involved in transforming growth factor-beta(TGF-β) and bone morphogenetic protein(BMP) [27]. In breast cancer patients, down-regulated miR-92a levels were found in tumor tissues and serum [28]. Dysregulated miR-127 in transgenic cloned sheep mediated the expression of retrotransposon-like gene 1(Rtl1), a key placental development gene that may cause developmental retardation [29]. MiR-694 was shown to be down-regulated in the liver of Lass II knockout mice and may be involved in the association between liver dysregulation and metabolism dysfunction [30]. MiR-375 over-expression was found to inhibit lung surfactant secretion, possibly by influencing actin dynamicand and injury pancreas in patients [31]. As tumor suppressors or oncogenes, the miR-310/13 cluster might regulate evolutionarily conserved immune signaling networks in cancer [32].

**Fig. 6:**
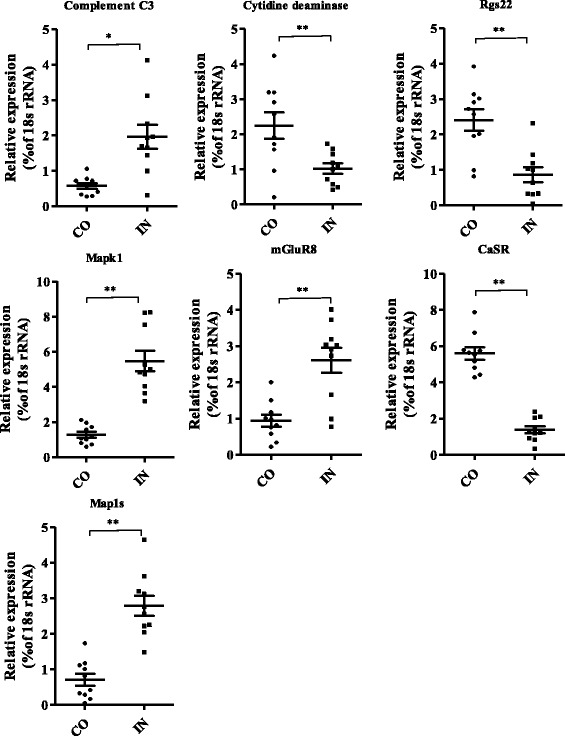
Quantitative real-time PCR validation of seven gene mRNA expression levels both the control group (CO) and infected group (IN). The expression levels of the indicated immune-regulated or signaling transmission genes in the CO and IN groups (*n* = 10 replicates per group) were normalized to that of 18 s rRNA. Statistical analyses were performed using unpaired Student’s t-tests. *P < 0.05 and **P < 0.01 by unpaired Student’s T-tests

Seven genes involved in immune-related responses were predicted to be the targets of seven miRNAs (miR-92d-3p, miR-127, miR-310-3p, miR-146-3p, miR-375-5p, miR-92, and miR-694). Complement C3 and cytidine deaminase were predicted as potential targets for miR-92d-3p and miR-127, respectively. The levels of complement C3 mRNA were reported to increase during the 24-h acute phase response of large yellow croaker (*Larimichthys crocea*) [33] and Miiuy croaker (*Miichthys miiuy*) [34] triggered by experimental infections. Up-regulation of complement C3 has been linked to innate immunity, which plays an important role in mediating tissue injury after stress. In this study, we found markedly up-regulated levels of complement C3 in the IN library (Fig. 6), suggesting its role as a potential modulator of the anti-inflammatory response defense against invading pathogens. Cytidine deaminase is a mutagenic activation-induced DNA/RNA-editing enzyme that has been implicated in human tumorigenesis. Abnormal cytidine deaminase levels were found in gastric epithelial cells infected by *Helicobacter pylori*, and it was suggested that this might be a mechanism responsible for the accumulation of submicroscopic deletions and somatic mutations [35]. Cytidine deaminase levels in aquatic animals have been little researched. Our results suggest that the down-regulated expression of cytidine deaminase mRNA after *S.iniae* infection for 24 h, may be associated with infectious diseases and immune-mediated disorders.

Regulators of G protein signaling (Rgs) are multi-functional GTPase-accelerating proteins that have become a focus of attention in recent years [36]. Down-regulated Rgs2 was reported in androgen-independent prostate tumor cells, which were inhibited by over-expression of Rgs2 in vivo and in vitro [37]. Rgs22 was a predicted target gene of miR-310-3p in our study. MiR-310-3p was down-regulated in the IN library (Fig. 6), suggesting that the reduction of Rgs22 may be associated with an increased inflammation response against infection. Signaling pathways of mitogen-activated protein kinases (Mapks) include extracellular signal-regulated kinase1/2 (ERK1/2), p38, cMYC, and c-Jun N-terminal kinase (JNK), which mediate the activities of several transcription factors [36]. Modulation of the Mapk signaling pathway may regulate the transcription of cell cycle genes, which was shown to be essential for mediating many cellular processes, including inflammatory response, cell stress, cell differentiation, and cell proliferation [38]. TNF-α is a major pro-inflammatory cytokine in cancer cells [39]. Meili et al.[39] and Zhu et al.[40] reported that TNF-α expression affected the downstream Mapk effector pathway. A significant increase in the expression of TNF-α mRNA was found in *S. iniae*-infected GIFT at 24 h post-infection [2], which might lead to activation of Mapk 1 and the immune response.

Metabotropic glutamate receptors (mGluRs) are widely known for their roles in synaptic signaling. Aberrant glutamate signaling has been shown to be involved in the transformation and maintenance for human cancer, including glioma, melanoma skin cancer, breast cancer, and prostate cancer [41]. In this study, mGluR8 was identified as a potential target of miR-375-5p, and mGluR8 mRNA was found to be up-regulated in the IN library, suggesting that mGluR8 may promote inflammatory cell infiltration via activation of the Mapk signaling pathways [42]. The calcium-sensing receptor (CaSR) is an important mediator of inflammation [43–45], and dysregulation of intestinal barrier integrity was reported to be induced in CaSR knockout mice [45]. Our findings suggest that down-regulation of CaSR mRNA expression may alter the composition of the gut microbiota and disrupt immune homeostasis in *S. iniae*-infected GIFT at 24 h post-infection.

Microtubule-associated protein 1S (Map1s) was the predicted target gene of miR-694. As a novel member of the microtubule-associatedprotein 1 (Map1) family, Map1s is important in inducing microtubule stabilization, bonding the microtubule organizing center to the centrosome, and regulation of mitotic cell cycle progression [46]. Zou et al. [47] reported that the interaction of suppressor of cytokine signaling 3 (SOCS3) and Map1s, and the integrity of the microtubule cytoskeleton was an important modulator of interleukin-6 (IL-6) signaling. Our results suggest that Map1s may be crucial in the response to pathogenic infection, and the regulation of Map1s by miR-694 may be involved in inflammatory regulating networks.

## Conclusions

In this study, we used high-throughput sequencing and analysis to screen for different miRNAs between normal and infected tilapia. We confirmed the expression levels of seven differentially expressed miRNAs and their predicted target genes, which may play essential roles in triggering the immune response of head kidney in *S. iniae*-infected GIFT at 24 h post-infection. These miRNAs and their putative target genes were associated mainly with autoimmune inflammation and regulation of cytokine signaling. Our findings provide insights into the role of miRNAs in the immune system of GIFT, and will help to form the basis for understanding miRNA-mediated control of gene expression against infection in GIFT. Further in vivo and invitro studies are needed to gain more insight into the association and the regulatory response between miRNAs and their predicted target genes, biological pathways, and molecular pathogenesis in tilapia.

## Methods

### Sample collection

Healthy adult male GIFT were obtained from the Yixing tilapia farm at the Freshwater Fisheries Research Center of the Chinese Academy of Fishery Sciences (Wuxi, China). The average weight of the fish was 350 ± 19 g. The fish were reared in indoor concrete tanks and acclimated for 2 weeks before treatment. During acclimation, the water temperature was maintained at 28 ± 0.3 °C and the fish were kept under a natural photoperiod and continuous aeration.

*S. iniae* bacteria (CMS No-005) were provided by the biotechnology laboratory at the Freshwater Fisheries Research Center of the Chinese Academy of Fishery Sciences [48]. The bacteria were inoculated into brain heart infusion (BHI) broth and incubated in a shaker (180 rpm) incubator at 28 °C overnight. The broth was centrifuged at 4000 *× g* for 5 min, washed twice with 0.65% sterile saline, We used turbidimetric methods to roughly estimate the stock solution concentration. We employed 10-fold serial dilutions to make five concentration gradients [10^5^; 10^6^; 10^7^; 10^8^ and 10^9^ colony forming unit (CFU) per ml] and determined the 96 h LD_50_. In the pre-experiment, 100 experimental fish were randomly distributed in five 800-L tanks each containing 20 fish. We injected different concentrations of bacteria intraperitoneally. The bacterial concentration was determined by plating 1 ml of the 10-fold serial dilutions onto BHI agar plates. In the formal experiment, the final concentration was 6.35 × 10^7^CFUmL^-1^. Fifty fish samples were assigned randomly to two groups of 25 samples each. One group was injected intraperitoneally with *S. iniae* at a concentration of 6.35 × 10^7^CFUmL^−1^ as the infected group (IN). We injected 300 μL/100 g fish weight of bacterial suspension into the abdominal cavity of the GIFT. The other group was injected with the same volume of 0.65% physiological saline as the control (CO). Fifteen fish from each treatment were anesthetized using 0.01% tricaine methanesulfonate and sacrificed 24 h after injection. The head kidney tissues were collected and immediately stored in liquid nitrogen at −80 °C for transcriptome sequencing and qRT-PCR.

### Construction of small RNA libraries by high-throughput sequencing

Total RNA was isolated from head kidney tissues using a miRNeasy kit (Takara, Dalian, China) according to the manufacturer’s protocol. The total RNA quantity and quality were determined using an Agilent 2100 Bioanalyzer (Agilent, Germany). We selected the samples with RNA integrity numbers more than 8.0 for further processing. The samples, which contained equal amounts of RNA extracted from the head kidney tissues of five fish (CO group or IN group), were mixed and pooled to construct the miRNA libraries. The two small RNA libraries were sequenced and built according to standard methods [49] at the Beijing Genomic Institute, Shenzhen, China (BGI, China).

### Analysis of basic sequencing data

Low quality reads and reads contaminated with adapter sequences were removed before analysis [11]. The filtered sequences between 18-nt and 30-nt long were aligned to the non-redundant animal miRNA sequences in miRBase (Release 21.0) (http://www.mirbase.org/), allowing no more than two mismatches outside the seed region. Reads that matched the miRNAs from other animals were considered to be conserved miRNAs in *O.niloticus*. Based on their similarity (identity score) to know miRNAs, we classified the conserved miRNAs into the corresponding miRNA families. The secondary structures of the remaining unidentified miRNAs were predicted using MIREAP (https://sourceforge.net/projects/mireap/) [50] and reads that could form the characteristic hairpin structure were classified as novel miRNAs.

### Identification of differentially expressed miRNAs

To determine the differential expression levels of the miRNAs between the CO and IN libraries, the sequencing data were normalized [51] and the fold-change was determined as: fold-change = log 2(IN/CO). The data from the two libraries were statistically analyzed based on the Audic and Claverie method [52].

### Prediction of target genes and bioinformatic analysis

We used TargetScan (http://www.targetscan.org/) and RNAhybrid (http://bibiserv.techfak.uni-bielefeld.de/rnahybrid) to predict the potential target genes of the differentially expressed miRNAs. The criteria used for target prediction were according to Zhang et al. [53]. We used the *O.niloticus* transcriptome sequence data (http://www.ncbi.nlm.nih.gov/genome/?term=Oreochromis%20niloticus) to screen and annotate immune-related target genes for further study. The GO (http://www.geneontology.org) and KEGG (http://www.genome.jp/kegg/pathway.html) databases were used to assign terms and pathways to the target genes to determine their potential downstream biological functions.

### Quantitative real-time PCR

The head kidney tissues from the 10 remaining fish in each group were used for qRT-PCR. A Mir-X™ miRNA First-Strand Synthesis kit (Takara, Dalian, China) was used to synthesize first-strand cDNA. The 10.0 μL reverse transcription (RT) reaction mixture contained 5.0 μL 2 × mRQ Buffer, 3.75 μL of the RNA sample (0.25-8 μg), and 1.25 μL of mRQ Enzyme. The reaction mixtures were incubated at 37 °C for 60 min, 85 °C for 5 min, followed by hold at 4 °C. The miRNA expression levels were determined using a miRNA SYBR Green qRT-PCR Kit (Takara, Dalian, China) with the provided miRNA universal primer (U6). The 25 μL PCR mixture included 2.0 μL of RT product, 12.5 μL 2 × SYBR Advantage Premix, 9 μL ddH_2_O, 0.5 μL 50× ROX Dye, 0.5μLmiRNA-specific Primer (10 μM), and 0.5 μL mRQ 3’ Primer. The reactions were performed at 95 °C for 10 s, followed by 40 cycles of 95 °C for 5 s, and 60 °C for 20s, with a final dissociation step at 95 °C for 60 s, 55 °C for 30s, and 95 °C for 30s. The miRNA specific primers (Table 2) were synthesized by Genewiz Inc., Suzhou, China.

**Table 2:** Primer design of miRNA

Name	Primer sequence (5’-3’)
miR-92	AATTGCACTCGTCCCGGC
miR-310-3p	TATGGCACTTCTCCCGGCCTG
miR-127	ATCGGATCCGTACTAGTGGTG
miR-599	GAAGTGTTACTGTGGAGGTCT
miR-92d-3p	TATGGCACTTATCCCGGCC
miR-146-3p	AGCGCTGTGGATCTTCGGTTTTC
miR-694	CTGAAAATGTATGGCGCTGGAG
miR-375-5p	ACTTGAGCGCGTGCGATAG

PrimeScript™ RT Master Mix (Takara, Dalian, China) was used for the RT reaction of the target genes. The 10.0 μL RT reaction mixture included 2.0 μL 5 × PrimeScript RT Master Mix, the RNA sample (≤500 ng), and RNase Free dH_2_O up to 10 μL. The reactions were incubated at 37 °C for 15 min, 85 °C for 5 s, followed by hold at 4 °C. The qRT-PCR was performed using SYBR® Premix Ex Taq kits(Takara, Dalian, China). The 20 μL PCR reaction mixture contained 2.0 μL of RT product, 10.0 μL 2 × SYBR® Premix Ex Taq II, 0.8 μL each of the forward and reverse primers (10 μM), 0.4 μL 50× ROX Dye, and 6 μL ddH_2_O. The reaction mixtures were incubated at 95 °C for 30 s, followed by 40 cycles of 95 °C for 5 s, and 60 °C for 30s. The primers are listed in Table 3. The transcript level of 18S rRNA was taken as the reference to calculate expression levels of the target genes. The miRNA and mRNA expression levels were analyzed using the 2^−ΔΔCt^ method [54], and the values related to the lower group represented the n-fold difference. The miRNA and mRNA expression levels were quantified using the ABI 7900HT Fast Real-Time PCR System (ABI, USA) and compared using the relative quantification (RQ) manager software. We used unpaired Student’s T-tests to analyze the qRT-PCR expression results.

**Table 3:** Primer design of putative target genes

Name	Primer sequence (5’-3’)
Complement C3	F: 5’-CAGGCAGGAGGATGTATCGG-3’ R: 3’- TGCCAGCGTCAAGTCTTTTCT-5’
Cytidine deaminase	F: 5’-CAGGCTGCAATGTGGAGAAT-3’ R: 3’-TGGTCCAACAAAGCGGTCTT-5’
Rgs22	F: 5’-TAGAGCGGCAGCGTTTGTG-3’ R: 3’-TGATCCACTGCATTCCTTGC-5’
Mapk1	F: 5’-TCAGCCCCTTTGAGCACC-3’ R: 3’-TGCACGGATAATGTCGTTGATT-5’
CaSR	F: 5’-AAAATCTATGATGCTTGTGGCTCC-3’ R: 3’-ATTGCCATGCAAGGGGAG-5’
Map1s	F: 5’-CTCCTCACTGGTCGCTAAAACT-3’ R: 3’-GGGACTGGTAGAAAGGGGTATGT-5’
mGluR8	F: 5’-TCAGCGAATCCCCACAGAC-3’ R: 3’-TGGGTAGCAGTTCGGGGTC-5’
18 s rRNA	F: 5’-GGCCGTTCTTAGTTGGTGGA-3’ R: 3’-TTGCTCAATCTCGTGTGGCT-5’

## Additional files

Additional file 1: Table S1. Cumulative mortality of GIFT at the injected dose of 10^5^; 10^6^; 10^7^; 10^8^ and 10^9^ CFU ml^-1^ respectively for 96 h. (DOCX 13 kb)
Additional file 2: Table S2. Statistics of the small RNA reads in the CO library. The GIFT were injected with the 0.65% physiological saline as the control (CO). The small RNA reads of CO group were analyzed and built by deep-sequencing. (DOCX 13 kb)
Additional file 3: Table S3. Statistics of the small RNA reads in the IN library. The GIFT were was injected intraperitoneally with *S. iniae* at a concentration of 6.35 × 10^7^CFUmL^−1^ as the infected group (IN). The small RNA reads of IN group were analyzed and built by deep-sequencing. (DOCX 13 kb)
Additional file 4: Table S4. Some of the miRNAs that were differentially expressed between the CO and IN libraries. Eight (miR-92/101c/310-3p/127/599/122-5p/10d-5p and let-7) were significantly up-regulated and 11 (miR-92d-3p/146-3p/17-5p/10/694/181b/138/10-5p/33a,/375-5p/10b) were significantly down-regulated in the IN library compared with the CO library based on deep-sequencing results. (DOCX 15 kb)

## Abbreviations

96 h LD_50_: 96 h semi-lethal *S. iniae* dose
BHI: Brain heart infusion
BMP: Bone morphogenetic protein
CaSR: Calcium-sensing receptor
CFU: Colony forming units
ERK1/2: Extracellular signal-regulated kinase1/2
GAP: GTPase-activating protein
GIFT: Genetically improved farmed tilapia
GO: Gene ontology
GPCR: G-protein coupled receptor
HECT: Homologous to E6AP C-terminus
HSP70: Heat-shock protein 70
IFN-γ: Interferon-γ
IL-1β: Interleukin-1β
IL-6: Interleukin-6
JNK: c-Jun N-terminal kinase
KEGG: Kyoto Encyclopedia of Genes and Genomes
LPS: Lipopolysaccharide
Map1s: Microtubule-associated protein 1S
Mapk: Mitogen-activated protein kinase
mGluR: Metabotropic glutamate receptor
miRNA: microRNA
NFIL3–6: Nuclear factor interleukin 3–6
qRT-PCR: Quantitative real-time PCR
Rgs: Regulators of G protein signaling
RQ: Relative quantification
RT: Reverse transcription
Rtl1: Retrotransposon-like gene 1
SMURF: E3 ubiquitin-protein ligase
SOCS3: Cytokine signaling 3
TGF-β: Transforming growth factor-beta
TLR4: Toll-like receptor4
TNF-α: Tumor necrosis factor-α
