# Supplementary material for: Effects of exposure to Streptococcus iniae on microRNA expression in the head kidney of genetically improved farmed tilapia (Oreochromis niloticus)
Source: BMC Genomics. 2017 Feb 20;18:190. doi: 10.1186/s12864-017-3591-z (PMC5322787; doi:10.1186/s12864-017-3591-z)
Supplement: Additional file 1: Table S1. — Cumulative mortality of GIFT at the injected dose of 105; 106; 107; 108 and 109 CFU ml-1 respectively for 96 h. (DOCX 13 kb) [file 12864_2017_3591_MOESM1_ESM.docx]

Table S1 Cumulative mortality of GIFT at the injected dose of 10^5^; 10^6^; 10^7^; 10^8^ and

| Injection dose | Fish | Infection time (h) | | | | |
| --- | --- | --- | --- | --- | --- | --- |
|  |  | 12 | 24 | 36 | 48 | 96 |
| 10^5^ | 20 | 100 | 100 | 95 | 80 | 75 |
| 10^6^ | 20 | 100 | 100 | 85 | 70 | 70 |
| 10^7^ | 20 | 100 | 90 | 65 | 60 | 55 |
| 10^8^ | 20 | 100 | 40 | 10 | 5 | 5 |
| 10^9^ | 20 | 100 | 0 | 0 | 0 | 0 |

10^9^ CFU ml^-1^ respectively for 96h
