# Supplementary material for: Effects of exposure to Streptococcus iniae on microRNA expression in the head kidney of genetically improved farmed tilapia (Oreochromis niloticus)
Source: BMC Genomics. 2017 Feb 20;18:190. doi: 10.1186/s12864-017-3591-z (PMC5322787; doi:10.1186/s12864-017-3591-z)
Supplement: Additional file 2: Table S2. — Statistics of the small RNA reads in the CO library. The GIFT were injected with the 0.65% physiological saline as the control (CO). The small RNA reads of CO group were analyzed and built by deep-sequencing. (DOCX 13 kb) [file 12864_2017_3591_MOESM2_ESM.docx]

Table S2 Read statistics of the obtained small RNAs of CO library

| Category | Unique sRNAs | Percent(%) | Total sRNAs | Percent(%) |
| --- | --- | --- | --- | --- |
| Total | 541895 | 100% | 12089630 | 100% |
| exon_antisense | 4858 | 0.9% | 54512 | 0.45% |
| exon_sense | 172460 | 31.83% | 305599 | 2.53% |
| intron_antisense | 5091 | 0.94% | 95504 | 0.79% |
| intron_sense | 31682 | 5.85% | 166591 | 1.38% |
| miRNA | 75522 | 13.94% | 8714764 | 72.08% |
| rRNA | 28693 | 5.29% | 1517831 | 12.55% |
| rRNAetc | 559 | 0.1% | 1068 | 0.01% |
| snRNA | 2119 | 0.39% | 11172 | 0.09% |
| snoRNA | 3685 | 0.68% | 24215 | 0.2% |
| tRNA | 7865 | 1.45% | 124482 | 1.03% |
| unann | 209361 | 38.63% | 1073892 | 8.88% |
