# Supplementary material for: Effects of exposure to Streptococcus iniae on microRNA expression in the head kidney of genetically improved farmed tilapia (Oreochromis niloticus)
Source: BMC Genomics. 2017 Feb 20;18:190. doi: 10.1186/s12864-017-3591-z (PMC5322787; doi:10.1186/s12864-017-3591-z)
Supplement: Additional file 3: Table S3. — Statistics of the small RNA reads in the IN library. The GIFT were was injected intraperitoneally with S. iniae at a concentration of 6.35 × 10^7^CFUmL−1 as the infected group (IN). The small RNA reads of IN group were analyzed and built by deep-sequencing. (DOCX 13 kb) [file 12864_2017_3591_MOESM3_ESM.docx]

| Category | Unique sRNAs | Percent(%) | Total sRNAs | Percent(%) |
| --- | --- | --- | --- | --- |
| Total | 640740 | 100% | 12624975 | 100% |
| exon_antisense | 5202 | 0.81% | 66510 | 0.53% |
| exon_sense | 213021 | 33.25% | 376264 | 2.98% |
| intron_antisense | 5096 | 0.8% | 110906 | 0.88% |
| intron_sense | 35816 | 5.59% | 218777 | 1.73% |
| miRNA | 76628 | 11.96% | 8124846 | 64.36% |
| rRNA | 35715 | 5.57% | 2023963 | 16.03% |
| rRNAetc | 576 | 0.09% | 1139 | 0.01% |
| snRNA | 2706 | 0.42% | 20389 | 0.16% |
| snoRNA | 4247 | 0.66% | 31547 | 0.25% |
| tRNA | 10811 | 1.69% | 205267 | 1.63% |
| unann | 250922 | 39.16% | 1445367 | 11.45% |

Table S3 Read statistics of the obtained small RNAs of IN library
