# Supplementary material for: Effects of exposure to Streptococcus iniae on microRNA expression in the head kidney of genetically improved farmed tilapia (Oreochromis niloticus)
Source: BMC Genomics. 2017 Feb 20;18:190. doi: 10.1186/s12864-017-3591-z (PMC5322787; doi:10.1186/s12864-017-3591-z)
Supplement: Additional file 4: Table S4. — Some of the miRNAs that were differentially expressed between the CO and IN libraries. Eight (miR-92/101c/310-3p/127/599/122-5p/10d-5p and let-7) were significantly up-regulated and 11 (miR-92d-3p/146-3p/17-5p/10/694/181b/138/10-5p/33a,/375-5p/10b) were significantly down-regulated in the IN library compared with the CO library based on deep-sequencing results. (DOCX 15 kb) [file 12864_2017_3591_MOESM4_ESM.docx]

Table S4 Differentially expressed miRNA between CO and IN of GIFT

| miR-name | CO-expressed | IN-expressed | CO-std | IN-std | fold-change  (log2 IN/CO) | sig |
| --- | --- | --- | --- | --- | --- | --- |
| miR-92d-3p | 3187 | 827 | 263.614 | 65.512 | -2.008 | ** |
| miR-17-5p | 606 | 224 | 50.126 | 17.743 | -1.498 | ** |
| miR-181b | 86159 | 22354 | 7126.686 | 1770.617 | -2.009 | ** |
| miR-146-3p | 12925 | 6681 | 1069.098 | 529.189 | -1.015 | ** |
| miR-10 | 160161 | 44971 | 13247.800 | 3562.067 | -1.895 | ** |
| miR-138 | 327 | 92 | 27.048 | 7.287 | -1.892 | ** |
| miR-146-3p | 160969 | 44912 | 13314.634 | 3557.393 | -1.904 | ** |
| miR-33a | 278 | 116 | 22.995 | 9.188 | -1.323 | ** |
| miR-375-5p | 13659 | 3127 | 1129.811 | 247.684 | -2.186 | ** |
| miR-694 | 7159 | 2000 | 592.160 | 133.000 | -2.155 | ** |
| miR-10b | 532619 | 1140945 | 44055.856 | 90372.060 | 1.037 | ** |
| miR-10d-5p | 20479 | 81456 | 1693.931 | 6451.973 | 1.929 | ** |
| miR-122-5p | 1870 | 4890 | 154.678 | 387.328 | 1.324 | ** |
| let-7 | 3500 | 1029 | 289.504 | 81.505 | 1.828 | ** |
| miR-310-3p | 653 | 3689 | 54.013 | 292.199 | 2.435 | ** |
| miR-599 | 41 | 121 | 3.391 | 9.584 | 1.546 | ** |
| miR-92 | 94823 | 680165 | 7843.334 | 53874.562 | 2.780 | ** |
| miR-101c | 20326 | 96801 | 1681.276 | 7667.421 | 2.189 | ** |
| miR-127 | 702 | 3353 | 58.066 | 265.585 | 2.197 | ** |

***P*<0.01.
